# Combination of Plasma Biomarkers and Clinical Data for the Detection of Myocardial Fibrosis or Aggravation of Heart Failure Symptoms in Heart Failure with Preserved Ejection Fraction Patients

**DOI:** 10.3390/jcm7110427

**Published:** 2018-11-08

**Authors:** Cho-Kai Wu, Mao-Yuan M. Su, Yi-Fan Wu, Juey-Jen Hwang, Lian-Yu Lin

**Affiliations:** 1 Division of Cardiology, Department of Internal Medicine, National Taiwan University College of Medicine and Hospital, Taipei 100, Taiwan; wuchokai@gmail.com (C.-K.W.); jueyhwang@ntu.edu.tw (J.-J.H.); 2 Department of Medical Imaging, National Taiwan University Hospital, Taipei 100, Taiwan; maoyuansu@gmail.com; 3 Department of Family Medicine, Taipei City Hospital Renai Branch, Taipei 106, Taiwan; geniuspenny@hotmail.com

**Keywords:** cardiac diastolic dysfunction, heart failure with preserved ejection fraction, biomarkers, cardiac magnetic resonance imaging, fibrosis

## Abstract

**Background:** Heart failure with preserved ejection fraction (HFpEF) is characterized by heart failure symptoms and structural change (including fibrosis). The relationship between novel biomarkers and the above components remains unclear. **Methods:** Seventy-seven HFpEF patients were recruited. All patients underwent echocardiography with tissue doppler imaging, cardiac magnetic resonance imaging (CMRI), and measurement of plasma inflammatory, remodelling, endothelial function, and heart failure biomarker levels. Myocardial fibrosis was defined by CMRI-extracellular volume. Forward conditional logistic regression was applied to demonstrate the determinants of myocardial fibrosis or heart failure symptoms. **Results:** The levels of growth differentiation factor, tissue inhibitor of metalloproteinase (TIMP)-1, galectin-3, and N-terminal pro b-type natriuretic peptide (NT-proBNP) were significantly higher in patients with more myocardial fibrosis. Matrix metalloproteinase-2 (MMP-2) and galectin-3 were independent markers of ECV. After adjusting for confounding factors, plasma galectin-3 and MMP-2 levels were correlated with myocardial fibrosis levels (odds ratio (OR): 1.05, 95% confidence interval (CI): 1.02 to 1.09, *p* = 0.005 and OR: 2.11, 95% CI: 1.35–3.28, respectively), while NT-proBNP level only was associated with heart failure symptoms. We developed a score system consisted of biomarkers and clinical parameters. The area under the curve of the scoring system receiver operating characteristic curve is 0.838 to predict the degree of myocardial diffuse fibrosis. **Conclusions:** In conclusion, we found that galectin-3 and MMP-2 were significantly associated with global cardiac fibrosis in HFpEF patients. We also combined plasma biomarkers and clinical data to identify HFpEF patients with more severe cardiac fibrosis.

## 1. Introduction

In recent years, population-based studies have indicated that the prevalence of heart failure with preserved ejection fraction (HFpEF) has been increasing [1]. The morbidity associated with HFpEF, especially the hospitalization rate, is proposed to be similar to that associated with systolic heart failure [2]. However, the treatment of this clinical entity has been limited because the mechanism of the disease is not yet fully understood. There are several possible mechanisms for the development of HEpEF, including dysfunction of myocardial calcium handling proteins, increased passive stiffness, endocardial and pericardial disorders, disproportional microvascular flow, and abnormal neurohormonal regulation [3]. One report suggested that the cardiac interstitium may play a pivotal role in the pathophysiology of HFpEF [4].

Although previous studies have shown that plasma biomarkers that reflect changes in extracellular matrix fibrillar collagen synthesis and degradation may predict the presence of HFpEF [5] and that biomarkers associated with myocardial fibrosis could be associated with major adverse cardiovascular events [6], the key plasma biomarkers associated with the degree of cardiac fibrosis remain unknown. Zile et al. examined various biomarkers and concluded that “fibrosis” plasma biomarkers have higher prediction rates for HFpEF than N-terminal pro–B-type natriuretic peptide (NT-proBNP) or clinical variables do. Therefore, it can be hypothesized that fibrosis biomarkers could actually reflect the severity of cardiac fibrosis, which is a major determinant of the symptoms or prognosis of HFpEF. Hence, it is crucial to discover the biomarkers associated with global myocardial fibrosis to improve the treatment, follow-up, and evaluation of HFpEF. Various biomarkers have been proposed to be associated with HFpEF [7,8]. For example, a biomarker associated with myocardial stress and stretch, NT-proBNP maintains prognostic value for composite of all-cause mortality and HF re-hospitalization at 1 year regardless of ejection fraction in a cohort study [7]. Biomarkers that associated with fibrosis, matrix metalloproteinases-2, for example, had also been proposed to have equal or better sensitivity and specificity than BNP in predicting HFpEF [7]. In addition, inflammatory biomarkers such as C-reactive protein, also have been reported to have stronger prognostic value for all-cause mortality and CV mortality in HFpEF than in HFrEF [7].

Owing to the importance of evaluating the extent of global cardiac fibrosis by non-invasive methods, several studies have focused on post-contrast cardiac magnetic resonance imaging (CMRI) myocardial T1 time as the gold standard to quantify diffuse myocardial fibrosis in patients with cardiomyopathies [9]. Nevertheless, this method is subject to variation and interference by the magnetic field used, acquisition timing, amount of contrast injected, and the renal function of the patients. In recent years, myocardial extracellular volume (ECV) adjusted by blood T1 time has been shown to be a more reliable method for identifying cardiac fibrosis especially for HFpEF patients [10,11].

We aimed to examine a variety of novel fibrosis and inflammatory biomarkers that has been proposed recently [7,8] in patients from the Taiwan Diastolic Heart Failure Registry (TDHFR) and to investigate the biomarkers associated with myocardial global fibrosis or those associated with aggravation of HF symptoms.

## 2. Materials and Methods

### 2.1. Study Participants and Study Design

The study group consisted of patients with heart failure admitted to the cardiovascular ward or outpatient clinics of the National Taiwan University Hospital from December 2011 to October 2015. Patients with a diagnosis of HFpEF (as defined in previous reports as well as by the 2007 consensus statement of the European Society of Cardiology) were enrolled from the TDHFR [12]. In brief, HFpEF was defined as: (1) heart failure defined according to the Framingham criteria and normal systolic function (ejection fraction ≥ 50%); and (2) echocardiographic evidence of left ventricular diastolic dysfunction: (1) a mitral inflow E/A ratio < 1, deceleration time > 220 cm/s, and decreased peak annular early diastolic velocity of the lateral mitral annulus < 8 cm/s in tissue doppler imaging (2) tissue doppler imaging E/e’ level more than 15 (3) 15 > E/e’ > 8 and left atrial volume index > 40 mL/m^2^ or left ventricular mass index > 122 g/m^2^ in female, >149 g/m^2^ in male. (4) 15 > E/e’ > 8 and NT-proBNP > 220 pg/mL. Signs or symptoms of congestive heart failure include lung crepitations, pulmonary oedema, ankle swelling, hepatomegaly, dyspnoea on exertion, and fatigue. Significant hepatic disease, secondary hypertension, pericardial disease, severe valvular heart disease, cancer, and chronic obstructive pulmonary disease were excluded [13,14]. All patients included in the current study underwent echocardiographic examinations, blood sampling for the examination of biomarkers and CMRI. This study was performed in accordance with the Declaration of Helsinki and was approved by the institutional review board of the National Taiwan University Hospital (approval ID: 20070313R), and all subjects provided their written informed consent prior to participation in the study. The methods were carried out in accordance with the approved guidelines. We evaluated the patients and compared their NYHA HF function class, medication history with their baseline condition (defined as a stable condition 3 months before the study of MRI). We also defined aggravation of HF symptoms including an increase in the NYHA functional class by at least one degree and/or new prescription of one or more additional diuretics for HF.

### 2.2. Measurement of Plasma Fibrosis and Inflammation Biomarkers

All blood samples were collected from each patient after 12 h of fasting. Biomarkers that were reported to be associated with HFpEF were examined. The blood was spun at 2000× *g* for 15 min, and then the plasma was separated and stored at −80 °C until use.

Levels of plasma inflammatory markers (tissue necrosis factor alpha (catalog no. HSTA00D; R & D Systems, 614 McKinley Place NE, Minneapolis, MN 55413, USA), interleukin-1 beta (catalog no. HSLB00C; R & D Systems, Minneapolis, MN, USA), interleukin-6 (catalog no. HST600B; R & D Systems, Minneapolis, MN, USA), C-reactive protein (catalog no. DCRP00. R & D Systems, Minneapolis, MN, USA), growth differentiation factor (GDF-15) (catalog no. DGD150, R & D Systems, Minneapolis, MN, USA) and osteopontin-A (catalog no. DOST00, R & D Systems, Minneapolis, MN, USA)), remodelling markers (tissue inhibitor of metalloproteinase 1 (TIMP1) (catalog no. DTM100, R & D Systems, Minneapolis, MN, USA), tissue inhibitor of metalloproteinase-2 (TIMP2) (catalog no. DTM200, R & D Systems, Minneapolis, MN, USA), matrix metalloproteinase-2 (MMP-2) (catalog no. MMP200, R & D Systems, Minneapolis, MN, USA), matrix metalloproteinase-9 (catalog no. DMP900, R & D Systems, Minneapolis, MN, USA), procollagen type I (cat no. MK101, Takara Bio Inc., Nojihigashi 7-4-38, Kusatsu, Shiga, Japan), galectin-3 (catalog no. DGAL30, R & D Systems, Minneapolis, MN, USA) and connective tissue growth factor (CTGF) (Cloud-Clone Corp, USCN life science Inc., 11271 Richmond Avenue, Suite H104, Houston, TX 77082, USA)), endothelial function markers (endothelin-1) (catalog no. DET100, R & D Systems, Minneapolis, MN, USA), and heart failure markers (suppression of tumorigenicity-2 (ST2) (catalog no. DST200, R & D Systems, Minneapolis, MN, USA) and N-terminal of the prohormone brain natriuretic peptide (NT-proBNP) (catalog no. BE6905796, IBL-America, 8201 Central Ave NE, Suite P, Minneapolis, MN 55432, USA)) were measured with high-sensitivity enzyme-linked immunosorbent assays.

### 2.3. Echocardiography

Left atrial (LA) diameter, left ventricular (LV) end diastolic diameter, systolic diameter, interventricular septum thickness, LV posterior wall thickness, mitral inflow early rapid filling wave (E), peak velocity of the late filling wave due to atrial contraction (A), E/A ratio, E wave deceleration time, and mitral annular early diastolic velocity were measured according to the American Society of Echocardiography’s guidelines by using an iE33 xMATRIX echocardiography system (Philips Healthcare, Best, The Netherlands). The peak annular early and late diastolic velocities of the lateral mitral annulus in tissue Doppler imaging (e’ and a’) were also recorded. Doppler and colour Doppler studies were performed to detect valvular heart disease. Significant valvular heart disease was defined as at least moderate aortic or mitral stenosis/regurgitation.

### 2.4. Magnetic Resonance Imaging Acquisition

MRI was performed on a 3-T MRI system (Trio, Siemens, Erlangen, Germany) with an 8-channel cardiovascular phased array torso coil. Myocardial T1 mapping was performed with an electrocardiography (ECG)-triggered Modified Look Locker Inversion recovery (MOLLI) sequence before and 10 min after a 0.15 mmol/kg intravenous administration of the gadolinium-based contrast agent (Omniscan, Winthrop Laboratories, GE Health care, Princeton, NJ, USA). The MOLLI protocol used two Look-Locker cycles to acquire seven images over 11 heart beats, and the scanning parameters were: TR/TE, 1.9 ms/1.0 ms; flip angle, 35°; minimum inversion time, 110 ms; inversion time increment, 80 ms; matrix size, 256 × 192; slice thickness, 6 mm; spatial resolution, 1.28 mm; GRAPPA acceleration factor, 2; number of inversions, 2; images acquired after first inversion, 5; pause 4 heart beats and images acquired after second inversion, 2. Five evenly spaced short-axis slices were acquired sequentially from the LV base to apex. After post-contrast T1 acquisition, late gadoninium enhancement (LGE) images were acquired using an ECG-triggered phase-sensitive inversion recovery prepared segmented fast gradient echo pulse sequence [15] at the same short-axis slices as those in the myocardial T1 mapping to identify focal fibrosis or scaring.

Cine MRI was performed using a segmented balanced steady-state gradient echo pulse sequence with a retrospective ECG R-wave trigger. The scanning parameters were: TR/TE, 3.0 ms/1.5 ms; flip angle, 46°; matrix size, 256 × 208 and spatial resolution, 1.21 mm. Multiple short-axis slices were obtained from the mitral orifice to the LV apex with a slice thickness of 8 mm and a gap of 2 mm. The true temporal resolution was 63 ms, and 30 cardiac phases were reconstructed retrospectively for each slice level.

### 2.5. Magnetic Resonance Imaging Analysis

Quantitative analysis of myocardial ECV was performed on T1 maps [8]. The regions of interest (ROIs) in the blood and the myocardium of the LV were drawn in the central area of the LV cavity and the septal myocardium on T1 maps for each slice, respectively. If the septal myocardium showed regional hyperenhancement on the LGE images, the ROI of the myocardium was re-drawn in the other unenhanced myocardial regions. The averaged T1 values of the segmented ROIs were then computed. After subtracting the pre-contrast values from the post-contrast values, the changes in the relaxation rate (1/T1) in the blood and in the myocardium were obtained. Myocardial ECV values were calculated using the ratio of the change in relaxation rate in the myocardium to that in the blood and multiplied by: (1-hematocrit). After excluding myocardium areas with LGE, we averaged each myocardial ECV value over five short-axis slices for each subject [16].

For LV function and mass analysis, endocardial and epicardial contours of the LV were determined at each slice level on cine MRI and the area enclosed by each contour was computed [17]. LV volumes for each time point were then determined by Simpson’s rule to obtain the volume-time curve of the LV. LV end diastolic volume (LVEDV) and LV end systolic volume (LVESV) were assessed from the volume-time curve for the maximal and minimal values and were used to compute LVEFs. LV mass was computed as the difference between LV epicardial volume at end-diastole and LVEDV, multiplied by the density of the myocardium, 1.05 g/cc. LV volumes and mass indexed to body surface area (BSA) were also measured from LVEDV (LVEDVi), LVESV (LVESVi), and LVM (LVMi) divided by BSA.

### 2.6. Statistical Analysis

Data were analysed using SPSS 15.0 software (SPSS Inc., Chicago, IL, USA). The normality of continuous variables was tested by Kolmogorov–Smirnov test. Since we found that most of the serum cytokine levels were not normally distributed, all the serum cytokine levels were expressed by median plus quartiles (25–75%). Variables with normal distribution were represented as mean values ± standard deviation, while categorical variables were represented as frequencies. Comparisons between continuous data with and without normal distribution were performed by using the Student’s *t* test and Mann–Whitney *U* test respectively while comparisons between categorical variables made using Pearson’s chi-square test. Multiple logistic regression modelling was applied followed by a forward stepwise analysis method to determine the factors associated with two outcomes CMRI fibrosis (ECV < 28% vs. ≥28%) and clinical heart failure. Factors including all the baselines, MRI, and laboratory parameters listed in Table 1 were used as independent variables. The median value of ECV (28%) was similar to that (27%) reported in a previous study as a cut point for normal and abnormal values [18]. The result of the logistic regression was also used to create a scoring system to predict abnormal ECV. The nearest integer of the odds ratio of each variable with significant correlation with the ECV was used as the “score” of that variable if the variable was over the cut point level. The cut point of a specific variable was determined by receiver operating characteristic curve (ROC) analysis with the largest area under curve (AUC) used to predict abnormal ECV. The total score was the sum of the score of each variable. The predictive ability of the scoring system was tested by calculating the area under curve (AUC) of the receiver operating characteristic curve (ROC). For all tests, a *p*-value < 0.05 was considered statistically significant.

## 3. Results

### 3.1. Baseline Characteristics and Echocardiographic Characteristics of Patients

A total of 77 patients with diastolic dysfunction were included in the study cohort. The baseline characteristics according to fibrosis (ECV value) severity are shown in Table 1. The baseline characteristics and comorbidities in both groups were comparable except for diabetes mellitus, indicating that this was an important factor for myocardial fibrosis. We also noted that patients with diastolic dysfunction with higher ECV had higher chances of experiencing symptoms of clinical heart failure (Table 1).

### 3.2. Plasma Levels of Biomarkers in Patients with Diastolic Dysfunction

Inflammatory, remodelling, endothelial, and heart failure markers were all examined. The detailed results of cytokines in each group were shown in Table 1. The levels of inflammatory marker plasma GDF-15 were significantly higher in patients with high-ECV than those in patients with low-ECV (high-ECV, 1297.50 (697.85–2543.90) pg/mL; low-ECV, 847.40 (625.50–1301.30); *p* = 0.018) (Table 1). Remodelling marker levels, including those of plasma MMP-2 and Galectin-3, were significantly higher in patients with high-ECV (MMP-2, high-ECV, 188.68 (149.79–254.82) ng/mL vs. low-ECV, 147.96 (131.61–184.50); *p* = 0.02; Galectin-3, high-ECV, 9.92 (7.70–15.41) ng/mL vs. low-ECV, 6.81 (5.59–8.67) ng/mL; *p* < 0.001). In addition, levels of NT-proBNP, the well-known heart failure marker, were also higher in the high-ECV group (high-ECV, 1530.0 (739.0–3150.0) pg/mL; low-ECV, 850.0 (439.5–1335.0); *p* = 0.021).

### 3.3. Factors (Biomarkers) Associated with Cardiac Fibrosis and Aggravated Heart Failure

The correlations among ECV and CMRI diastolic and systolic function parameters and biomarkers were assessed. We then applied a forward stepwise analysis method to determine the factors associated with ECV. The results are demonstrated in Table 2. Diabetes mellitus was a traditional risk factor associated with cardiac fibrosis in this cohort. MMP-2 and Galectin-3 levels were associated with higher rates of ECV (odds ratio (OR): 1.05, 95% confidence interval (CI): 1.02 to 1.09, *p* = 0.005 and OR: 2.11, 95% CI: 1.35–3.28, respectively)), whereas endothelin-1 had a borderline relationship with the development of higher cardiac fibrosis (OR: 16.207, 95% CI: 0.961–273.333, *p* = 0.053). For the determinants of aggravated heart failure, we performed another forward stepwise analysis and found that TIMP1 and NT-proBNP were the major determinants for the presence of aggravated heart failure (OR: 1.025, 95% CI: 1.001–1.050 and OR: 1.010, 95% CI: 1.003–1.017, respectively).

**Figure 1:**
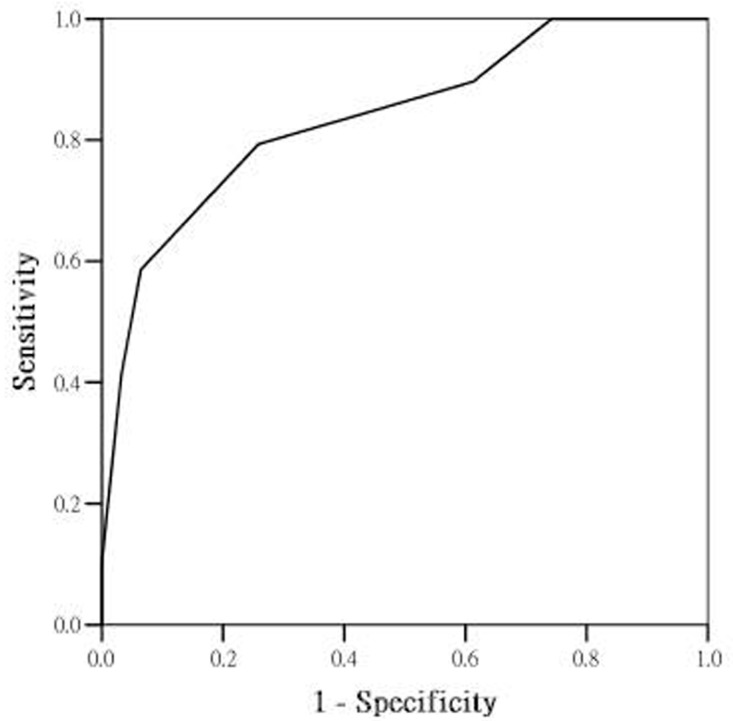
Receiver operating characteristic curve (ROC) of the scoring system used to predict the degree of myocardial diffuse fibrosis. The area under curve is 0.838.

We created a scoring system by using diabetes mellitus status (yes = 2, no = 0) and the median levels of MMP-2 (2 vs. 0), galectin-3 (1 vs. 0) and endothelin-1 (2 vs. 0), as cut-off points. The results of this scoring system are presented in Table 3. The ROC for ECV is shown in Figure 1. The AUC of the scoring system was 0.838 (0.737–0.939).

## 4. Discussion

In the current study, we evaluated all patients with HFpEF by using CMRI and determined the global absolute cardiac fibrosis value. We were able to identify the extent of fibrosis in each patient with HFpEF and measured a variety of inflammation, cardiac remodelling, endothelial function, and heart failure cytokine levels. We found that galectin-3 and MMP-2 were significantly associated with global cardiac fibrosis, even after adjusting for confounding risk factors, whereas TIMP2 and NT-proBNP were significantly associated with the aggravation of heart failure symptoms. We also developed a cytokine model along with inclusion of clinical parameters to identify HFpEF patients with more severe cardiac fibrosis. To the best of our knowledge, this is the first report to demonstrate and differentiate the relationship between a variety of cytokines and cardiac ECV (fibrosis content) or heart failure symptoms in a prospective HFpEF cohort. With our models, a clinical physician could decide the level of fibrosis and actual severity of HFpEF patients by blood tests and clinical history.

### 4.1. Major Findings

We found that NT-proBNP level was unable to differentiate the degree of fibrosis well, and that it was significantly associated with heart failure symptoms. One possible explanation for this is that NT-proBNP is a marker of volume overload rather than of cardiac dysfunction [19]. Previous studies showed that NT-proBNP level can indicate diastolic dysfunction in patients with HFpEF with moderate tissue Doppler E/Ea ratios [20]. NT-proBNP levels are also related to atrial volume enlargement, which is considered an indirect marker of filling pressure [21,22]. In patients with HFpEF, increased NT-proBNP level is directly related to increased LV diastolic filling pressure and end diastolic wall stress [23]. NT-proBNP level is more closely related to LV diastolic wall stress. Therefore, NT-proBNP level decreases when LV diastolic pressure decreases in response to volume reduction [24]. Hence, in patients with LVDD who have a smaller LV cavity size with much lower end diastolic wall stress than that in patients with HFpEF, even in the context of high diastolic pressures, a lower stimulus for the production of NT-proBNP is present. In this context, NT-proBNP level may correlate with the development of heart failure symptoms, but not with the actual extent of fibrosis or possible long-term outcomes in patients with LVDD. In addition, TIMPs have multiple mechanisms of action and targets for activity. Generally, TIMPs may bind to active MMPs and inactivate their protease activity, leading to a reduction in collagen degradation. Alternatively, TIMPs may affect myocardial growth, fibroblast proliferation, and activity. Increasing TIMPs levels correlate with increased fibroblast growth factors and can stimulate profibrotic signalling cascades and contribute to fibrosis [24,25]. In the present cohort, TIMPs levels were significantly higher in patients with greater degrees of myocardial fibrosis (TIMP1, Table 1), which could reflect changes in collagen homeostasis. Patients with increasing TIMPs levels also had significant manifestations of heart failure symptoms after adjusting for potential confounding factors (Table 2). TIMPs may represent the transition from antecedent diseases like hypertension to clinically symptomatic heart failure. The patients in this cohort did not suffer from obvious LV hypertrophy, and LVDD mainly resulted from myocardial fibrosis. TIMPs control upstream myocardial collagen turnover and may, therefore, be associated with both heart failure symptoms and the severity of myocardial fibrosis in this cohort. In addition, our current scoring system incorporated clinical parameters and biomarkers to provide precise prediction for the severity of myocardial fibrosis (Figure 1). Current prevalent biomarkers (ST2, NT-proBNP, etc.) mostly predicted the fluid status of HFpEF patients but cannot predict actual fibrotic severity for the patients. Using this new scoring system could help clinical physicians to differentiate those with advanced-stage (more fibrotic) HFpEF by a simple blood test. For those with higher scores, we realized that these patients are fragile and could develop severe symptoms with little fluid status change. Therefore, strict fluid limitation, blood pressure control, or even aggressive therapies should be performed even if the patients remain asymptomatic or the NT-proBNP level is within normal range.

We calculated the degree of diffuse cardiac fibrosis by CMRI-ECV, which should not be affected by confounding factors such as magnetic field, acquisition timing, the amount of contrast injected, and the renal function of patients. CMRI-ECV has been shown to be stable over a wide time range after contrast administration [10]. Galectin-3 and MMP-2 correlate to CMRI-ECV even after adjusting for other comorbidities. Galectin-3 is secreted by activated macrophages and modulates several physiological and pathological processes that are associated with the development of HFpEF, including inflammation and fibrosis [26]. Our previous studies concluded that mechanical stretching increased galectin-3 secretion in cultured cardiomyocytes, and both plasma and myocardial galectin-3 levels were found to be correlated with the severity of cardiac diastolic dysfunction. We also provided evidence for the possibility that the myocardium could secret galectin-3 under the stimulation of myocardial stretching (or pressure overload), and that the secreted galectin-3 may in turn trigger myocardial fibrosis, resulting in LV diastolic dysfunction [27]. Furthermore, the myocardial extracellular matrix (ECM) plays a critical role in cardiac architecture and function [28]. ECM balance is regulated by complex interactions between MMPs, which degrade collagen and other ECM components, and their specific tissue inhibitors. Checking plasma MMP levels may be a method for quantifying collagen turnover in HFpEF, with implications for assessing disease severity, prognosis, and responses to treatment. Compared to more thoroughly investigated MMPs, increases in MMP-2 levels have been reported most consistently in patients with HFpEF [5,29]. Hence, in our current cohort of patients with LV dysfunction, only MMP-2 had a significant correlation with global cardiac fibrosis. Therefore, biomarkers representative of true cardiac fibrosis such as galectin-3 and MMP-2 may reflect the severity of diastolic dysfunction more stably, while biomarkers associated with diastolic filling pressure (e.g., NT-proBNP) reflect current disease status or the severity of acute heart failure symptoms. BNP has been shown to be a strong predictor of short-term outcomes or symptoms in patients with HFpEF [30,31], but patients with HFpEF with higher galectin-3 or MMP-2 levels had poorer long-term prognoses [32].

Various studies have assessed biomarkers and HFpEF. Most studies examined single biomarkers or groups of similar biomarkers, and thus the results were often inconsistent. A recent study included a group of patients with HFpEF from the PARAMOUNT trial and compared the predictive value of four biomarkers (ST2, galectin-3, MMP-2, and collagen III N-terminal propeptide). The authors concluded that in patients with HFpEF, the levels of biomarkers that reflect collagen homeostasis were correlated with the severity of disease and underlying pathophysiology and may modify the structural response to treatment [33]. The most important components for diastolic function in heart failure are chamber stiffness and relaxation. Efficient diagnostic and prognostic biomarkers remain undetermined in this population. Biomarkers that mostly extensively examined in HFpEF are extracellular matrix or remodelling biomarkers. Zile et al. examined a panel of remodelling biomarkers along with clinical variables in a group of left ventricular hypertrophy patients and those who have development of HFpEF [5]. A plasma multibiomarker panel consisting of increased remodelling biomarkers (MMPs, TIMPs) and NT-proBNP predicted the presence of left ventricular hypertrophy well. The authors then concluded that plasma biomarkers reflecting changes in extracellular matrix fibrillar collagen homeostasis have discriminative value in identifying the presence of HFpEF, possibly better then clinical indices. Furthermore, according to a recent review and our previous studies, several biomarkers, including biomarkers of myocyte stress, inflammation, GDF-15, cystatin C, and galectin-3, were associated with development of HFpEF and even with clinical outcomes of HFNEF patients in terms of morbidity and mortality [13,27,34]. Besides, fibrosis in HFpEF is closely linked to inflammation. Histological study for HFpEF myocardium revealed an increased collagen volume fraction, higher expression of collagen type I, and more collagen cross-linking, which all contributed to diastolic LV dysfunction, which was in agreement with our cohort [35]. The authors proposed that myocardial collagen deposition might result from differentiation of fibroblasts into myofibroblasts induced by inflammation released by monocytes [36]. These inflammations could reduce NO bioavailability and increase the function of profibrotic action of endothelin-1 or angiotensin II [37]. Therefore, inflammatory response could stimulate cardiac diastolic dysfunction initially [13] and could result in myocardial fibrosis [38].

To summarize the results from numerous studies and a review article, myocardial fibrosis could be resulted from multiple pathways and unlike most previous studies [39,40], we evaluated modern biomarkers from all aspects, including inflammatory, collagen turnover, and heat failure indicators. The goal of this project was to test the discriminative value of a large portfolio of candidate biomarkers from multiple pathways in HFpEF patients. All patients included in this study underwent CMRI-ECV examination, and we were able to assess the degree of LV fibrosis precisely. Therefore, we provide evidence in support of the concept above that only biomarkers related to collagen turnover (galectin-3, MMP-2) were associated with structural changes and could be targets for further therapy or predictors of treatment response.

### 4.2. Study Limitations

The present study had several limitations. First, this was a cross-sectional study, and the number of patients included was small. Further large-scale studies are warranted to determine the prognostic value cytokine levels in patients with HFpEF. Second, the findings were purely correlative, and are not sufficient to indicate any causative relationships. Third, we did not directly measure tissue biomarker expression in patients with HFpEF. Theoretically, this is not feasible in human studies. However, we successfully measured the severity of tissue fibrosis by using CMRI-ECV and confirmed the presence of correlations between various novel cytokines and fibrosis or heart failure symptoms. We believe that CMRI-ECV is the most reliable non-invasive method for elucidating the relationships between cytokines and tissue or clinical expression.

In conclusion, we successfully studied the associations between plasma biomarkers with myocardial fibrosis or heart failure symptoms in a cohort of HFpEF patients. We found a significant correlation among plasma NT-proBNP, TIMP, and the development of heart failure symptoms. We also demonstrated that galectin-3 and MMP-2 levels increased according to the severity of myocardial fibrosis after adjusting for confounding factors. The results of this study indicated that different biomarkers could be important intermediaries in promoting further fibrosis and/or inflammatory cascades, or may reflect the elevation of cardiac diastolic filling pressure. While there is little information available regarding the medical management of HFpEF, novel therapies that downregulate the expression of these biomarkers may represent a new direction. Through examination of cytokine levels, the status, aetiology, or severity of cardiac fibrosis and LV diastolic dysfunction can be determined. This may allow more individualized therapies to be developed in the future.

## Figures and Tables

**Table 1:** Comparison of basic characteristics, risk factors, comorbidities, medications, and serum biomarkers between patients with low and high ECV in the study group.

	ECV < 28% (*n* = 39)	ECV ≥ 28% (*n* = 38)	*p*-Value
Baseline			
Age (mean ± SD)	67.2 ± 10.4	68.4 ± 12.6	0.632
Sex, M (%)	51.3	36.8	0.202
Risk factors			
HTN (%)	84.6	78.9	0.519
Dyslipidemia (%)	48.7	42.1	0.560
Comorbidities			
CAD (%) PCI (%) CABG (%)	41.0 41.0 0.0	36.8 36.8 5.3	0.707 0.707 0.240
Stroke	2.6	5.3	0.615
PAD (%)	5.1	2.6	0.552
Previous MI (%)	7.7	5.3	1.000
CKD (%)	2.6	7.9	0.358
eGFR, mL/min/1.73 m^2^ (mean ± SD)	78.39 ± 18.76	70.21 ± 17.96	0.054
Aggravation of HF (%)	28.2	52.6	0.029 *
Medications			
Anti-platelet (%)	28.2	36.8	0.418
ACEI (%)	0.0	2.6	0.494
ARB (%)	53.8	52.6	0.915
MRI parameters			
LVEDVi, mL/m^2^ (mean ± SD)	48.43 ± 12.15	51.37 ± 10.70	0.265
LVESVi, mL/m^2^ (mean ± SD)	10.25 ± 5.16	11.25 ± 6.15	0.444
LVMi, g/m^2^ (mean ± SD)	59.76 ± 11.71	61.63 ± 13.86	0.525
LVEF, % (mean ± SD)	78.54 ± 7.11	77.80 ± 9.01	0.723
Inflammatory markers **			
TNF-α, pg/mL	1.98 (1.65–2.51)	1.80 (1.58–2.16)	0.300
IL-1β, pg/mL	0.44 (0.34–0.62)	0.34 (0.22–0.47)	0.108
IL-6, pg/mL	1.68 (1.12–2.39)	1.51 (1.16–3.34)	0.756
CRP, ug/mL	1.31 (0.77–2.45)	1.27 (0.79–3.64)	0.395
GDF15, pg/mL	847.40 (625.50–1301.30)	1297.50 (697.85–2543.90)	0.018 *
OPN, ng/mL	106.37 (83.39–124.51)	101.90 (84.62–130.91)	0.965
Remodeling markers **			
TIMP1, ng/mL	112.94 (98.75–123.96)	124.58 (96.21–163.48)	0.081
TIMP2, ng/mL	130.00 (108.90–147.60)	144.90 (85.85–183.33)	0.190
MMP-2, ng/mL	147.96 (131.61–184.50)	188.68 (149.79–254.82)	0.020
MMP-9, ng/mL	178.40 (116.00–294.30)	159.95 (88.38–449.0)	0.815
PIP, ng/mL	202.02 (158.64–236.55)	202.86 (162.05–300.74)	0.492
Galectin-3, ng/mL	6.81 (5.59–8.67)	9.92 (7.70–15.41)	<0.001 *
CTGF, ng/mL	0.11 (0.00–1.97)	0.07 (0.00–13.81)	0.704
Endothelial function **			
Endothelin-1, pg/mL	0.96 (0.80–1.24)	1.15 (0.88–1.34)	0.203
Heart failure markers **			
NT-proBNP, pg/mL	850.0 (439.5–1335.0)	153.00 (739.0–3150.0)	0.021 *
ST2, ng/mL	8.79 (6.71–13.52)	8.96 (7.17–12.95)	0.951
DM (%)	12.8	44.7	0.002 *

Abbreviations: BSA, body surface area; HTN, hypertension, defined as blood pressure greater than 140/90 mmHg or use of antihypertensive drug; DM, diabetes mellitus; CKD, chronic kidney disease, defined as eGFR < 60 mL/min/1.73 m^2^; Dyslipidemia, defined as high density lipoprotein < 40 mg/dL or low density cholesterol > 130 mg/dL or triglyceride > 150 mg/dL or use of statins/fibrates; HF, heart failure; eGFR, estimated glomerular filtration rate; CAD, coronary artery disease, defined as a coronary artery stenosis > 50%; PCI, percutaneous coronary intervention; CABG, coronary artery bypass graft; MI, myocardial infarction; PAD, peripheral arterial occlusive disease; ECV, extracellular volume fraction; ACEI, angiotensin converting enzyme inhibitor; ARB, angiotensin receptor blocker; MRI, Magnetic Resonance Imaging; ECV, extracellular volume fraction; LVEDVi, left ventricular end-diastolic volume index; LVESVi, left ventricular end-systolic volume index; LVMi, left ventricular mass index; LVEF, left ventricular ejection fraction; TNF-α, tissue necrosis factor alpha; IL-1β, interleukin-1 beta; IL-6, interleukin-6; CRP, C-reactive protein; GDF-15, growth differentiation factor; OPN, osteopontin-A; TIMP1, tissue inhibitor of metalloproteinase 1; TIMP2, tissue inhibitor of metalloproteinase-2; MMP-2, matrix metalloproteinase-2; MMP-9, matrix metalloproteinase-9; PIP-1, procollagen type I; CTGF, connective tissue growth factor; NT-proBNP, N-terminal of the prohormone brain natriuretic peptide; ST2, suppression of tumorigenicity-2. * *p* < 0.05; ** All biomarker levels were shown as median plus (quartiles 25–75%).

**Table 2:** Logistic regression analysis demonstrating the determinants of ECV and heart failure.

	OR	95% CI	*p*-Value
ECV ^a^			
DM	10.732	1.542–74.696	0.017
MMP-2	1.053	1.016–1.091	0.005 *
Galectin-3	2.105	1.352–3.277	0.001 *
Endothelin-1	16.207	0.961–273.333	0.053
Heart failure ^b^			
TIMP2	1.025	1.001–1.050	0.044 *
NT-proBNP	1.010	1.003–1.017	0.004 *

Forward conditional logistic regression; ^a^ Cox and Snell R square: 0.534; ^b^ Cox and Snell R square: 0.393; Abbreviations: DM, diabetes; MMP-2, matrix metalloproteinase-2; TIMP2, tissue inhibitor of metalloproteinase-2; NT-proBNP, N-terminal of the prohormone brain natriuretic peptide; OR, odds ratio; CI, confidence interval; ECV, extracellular volume fraction. * *p* < 0.05.

**Table 3:** Scoring system and area under curve (AUC) to predict the degree of myocardial diffuse fibrosis by using median values as cut-off points.

	Cut Point	OR (95% CI)	Beta Coefficient	Score	AUC (95% CI)
DM	Yes	7.808 (1.654–36.854)	2.055	2	0.660 (0.536–0.783)
MMP-2	≥164.75 ng/mL	5.654 (1.321–24.128)	1.731	2	0.636 (0.511–0.761)
Galectin	≥8.32 ng/mL	4.413 (1.141–17.075)	1.485	1	0.662 (0.540–0.785)
Endothelin-1	≥1.02 pg/mL	5.681 (1.389–23.238)	1.737	2	0.617 (0.474–0.760)
Combine				0–7	0.838 (0.737–0.939)

Abbreviations: DM, diabetes; MMP-2, matrix metalloproteinase-2; OR, odds ratio; CI, confidence interval.
